# Cancer incidence and mortality in Bucaramanga, Colombia. 2008-2012

**DOI:** 10.25100/cm.v49i1.3632

**Published:** 2018-03-30

**Authors:** Claudia Janeth Uribe Pérez, Claudia Milena Hormiga Sánchez, Sergio Eduardo Serrano Gómez

**Affiliations:** 1 Grupo de investigación Estudio Genético de Enfermedades Complejas, Universidad Autónoma de Bucaramanga. Bucaramanga, Colombia.; 2 Directora del Registro Poblacional de Cáncer del Área Metropolitana de Bucaramanga. Bucaramanga, Colombia.; 3 Grupo de investigación Observatorio de Salud Pública de Santander, Fundación FOSCAL- Universidad Autónoma de Bucaramanga. Bucaramanga, Colombia; 4 Grupo de Investigaciones Clínicas UNAB, Universidad Autónoma de Bucaramanga. Bucaramanga, Colombia

**Keywords:** cancer, incidence, mortality, records as subject, Bucaramanga, cáncer, incidencia, mortalidad, registros como asunto, Bucaramanga

## Abstract

**Introduction:**

Cancer is a burden in the world, especially for the least developed countries. The Population Registries of Cancer are fundamental in order to know the territorial profiles of cancer, and to evaluate the impact of their control programs.

**Objective:**

To estimate the incidence and mortality from cancer in the Metropolitan Area of ​​Bucaramanga in the period 2008-2012.

**Methods:**

A descriptive population study of cancer incidence and mortality in the Metropolitan Area of ​​Bucaramanga was conducted. Primary invasive cancer cases from the 2008-2012 period was obtained from the RPC-AMB base. Population and death data were provided by the National Administrative Department of Statistics (DANE, for its initials in Spanish). Crude rates of global and specific incidence and mortality were estimated by sex, and standardized incidence and mortality rates.

**Results:**

During the five-year period, 8,775 incidents of cancer were recorded (excluding non-melanoma skin cancer). The global standardized incidence rates per 100,000 person-years were 151.7 in men and 157.2 in women. The main locations were prostate, stomach and colorectal, in men; breast, thyroid and colorectal in women. The standardized mortality rate per 100,000 person-years was 94.8 in men and 78.0 in women.

**Conclusion:**

The incidence and mortality rates in most locations are lower than the national ones and those in the previous quinquennium in the Metropolitan Area of ​​Bucaramanga. Thyroid cancer, colorectal cancer, and leukemia show a tendency to increase, which demands further investigation.

## Resumen

**Introducción:**

El cáncer constituye una carga en el mundo, en especial para los países menos desarrollados. Los Registros Poblacionales de Cáncer son fundamentales para conocer los perfiles territoriales del cáncer y la evaluación del impacto de sus programas de control.

**Objetivo:**

Estimar la incidencia y mortalidad por cáncer en el Área Metropolitana de Bucaramanga en el período 2008-2012.

**Métodos:**

Se realizó un estudio poblacional descriptivo de la incidencia y mortalidad por cáncer en el Área Metropolitana de Bucaramanga. Los casos de cáncer invasivos primarios del periodo 2008-2012 se obtuvieron de la base del RPC-AMB. Los datos de población y defunciones fueron facilitados por el Departamento Administrativo Nacional de Estadística (DANE). Se estimaron tasas crudas de incidencia y mortalidad globales y específicas por sexo, y tasas de incidencia y mortalidad estandarizadas.

**Resultados:**

Durante el quinquenio se registraron 8,775 casos incidentes de cáncer (excluyendo cáncer de piel no melanoma). Las tasas de incidencia estandarizada globales por 100,000 personas-año fueron de 151.7 en hombres y 157.2 en mujeres. Las principales localizaciones fueron próstata, estómago y colorrecto, en los hombres, y mama, tiroides y colorrecto en las mujeres. La tasa de mortalidad estandarizada por 100,000 personas-año fue de 94.8 en hombres y de 78.0 en mujeres.

**Conclusión:**

Las tasas de incidencia y mortalidad en la mayoría de localizaciones son inferiores a las nacionales y al quinquenio previo en el Área Metropolitana de Bucaramanga. El cáncer de tiroides, colorrectal, y las leucemias muestran una tendencia al aumento, lo cual demanda indagaciones posteriores.

## Introduction

Cancer represents a huge burden in the world, causing about 14.1 million new cases and 8.2 million deaths in 2012. The burden of cancer is increasing in less developed countries, from which about 57% of the new cases come up as well as 65% of the deaths caused by this disease. It is estimated that 48% of prevalent cases of cancer at five years of diagnosis occur in the regions with less economic development ^1^ ^,^ ^2^.

The incidence of cancer is increasing. It is expected that by 2025, there will be more than 20 million new cases, and a greater burden in low and middle income countries ^3^ ^,^ ^4^. This trend is attributed to the growth and aging of the population, as well as to a growing prevalence of clearly established risk factors, such as smoking, overweight, physical inactivity and changes in reproductive patterns associated with urbanization and economic development ^1^ ^,^ ^2^.

To generate impact on the reduction of the problems generated by this disease, the World Health Organization (WHO) promotes the creation of effective programs that improve early detection, timely diagnosis, and a specific and effective treatment ^5^. 

It also leads cancer control programs with the promotion of national cancer control policies, plans and programs. One of the strategies has been the promotion, establishment and strengthening of the monitoring and evaluation of this disease through registries, as well as research aimed at the burden of the disease, as well as increasing the availability of resources for its care ^6^.

Population Cancer Registries (RPC, for its initials in Spanish) are essential information systems for cancer control programs; these allow determining frequencies, incidences, mortality and trends, which are the result of activities aimed at collecting, analyzing and continuously disseminating information on cancer cases that occur in a specific population, with guarantee of the quality of the data (completeness, precision, and comparability) ^7^ ^,^ ^8^.

In Colombia there are four RPC with international recognition. One of these is the Population Registry of Cancer of the Metropolitan Area of ​​Bucaramanga (RPC-AMB), which has managed to consolidate a continuous work during 18 years of operation ^9^ ^,^ ^10^.

With the leadership of the Ministry for Health and Social Protection, the National Cancer Information System was set up and the National Cancer Observatory for Colombia was created, led by the National Cancer Institute of Colombia (INC, for its initials in Spanish), in order to know the territorial profile and evaluate the impact of cancer control programs ^11^. Population Cancer Registries are a fundamental part of this initiative ^12^; through them, the epidemiology of the disease can be known; and also the rate progress is being made in terms of fulfilling one of the pillars of the Ten-Year Plan for Cancer Control ^13^.

This study describes the total and specific incidence and mortality rates by sex, corresponding to the Metropolitan Area of ​​Bucaramanga during the period 2008-2012.

## Materials and Methods

A descriptive population study was proposed, with the incident cases of invasive cancer registered in the databases of the RPC-AMB in the quinquennium 2008-2012.

### Geographic area

Metropolitan Area of ​​Bucaramanga is a geographical area made up of four municipalities in the province of Santander: Bucaramanga (its capital city), Floridablanca, Girón and Piedecuesta. It is located in the Andean region and it has an area of ​​1,479 km^2^. Each municipality is independent in its political organization, but they are completely related from an economic and social perspective ^14^.

According to population projections of DANE, AMB had 1,074,000 inhabitants at the middle of the 2008-2012 period (53.5% of the provincial total); 94.3% were urban residents, 51.9% were women, 24.5% were aged under 15 years, and 20.4% were older than 50 years ^15^.

AMB is an important reference center for the care of cancer patients in the northeastern part of the country, with state (hospitals) and private institutions (clinics, specialized medical centers, diagnostic centers, oncology centers and medical specialists in all areas) that provide various oncological services for diagnosis and treatment.

### The Population Registry of Cancer of the Metropolitan Area of ​​Bucaramanga (RPC-AMB)

RPC-AMB has carried out permanent activities since April 2000, as a result of the commitment assumed by La Universidad Autónoma de Bucaramanga)**.** The economic resources are assigned in the research calls of the university, and it receives annual co-financing from the INC.

### Method of data collection and processing

RPC-AMB makes active collection of incident cases through periodic visits to information sources: pathology and hematology laboratories, hospital discharges, imaging centers, screening centers, volunteer programs, oncology centers, medical specialists, autopsies and certificates of deaths that are obtained in the Provincial Health Secretariat. 

A recordable case is any malignancy of any location including benign neoplasms of the Central Nervous System (CNS) and carcinomas in situ that have been diagnosed after January 1 of the year 2000 in residents of AMB, both in urban and rural areas, regardless of the diagnostic method used, so that cases identified by death certificate are also included. It excludes non-melanoma skin cancer (basal cell and squamous cell).

The cases obtained by hospital discharge and death certificates follow a process of verification of the diagnosis, through the search of the clinical history. When no additional information is obtained from the case obtained by the death certificate, it is recorded as a case identified only by its death certificate (death certificate only - DCO).

The coding of cancer cases is carried out under the supervision of the RPC Director, by personnel trained in the International Classification of Diseases for Oncology (ICD-O) - 3rd edition, 1st revision, published by the World Health Organization (WHO) ) in 2013, and the IARC criteria for multiple primary tumors ^16^ ^,^ ^17^.

RPC-AMB complies with confidentiality standards following the parameters of the IARC and the research commitment ethics. Only the staff of RPC-AMB accesses the information of each case, which allows exhaustive work in the control of the duplicity of the information ^8^ ^,^ ^18^.

The source of information on deaths was DANE, the official entity that collects, organizes and codifies the basic causes of mortality using the tenth revision of the International Classification of Diseases (ICD 10) ^19^.

The CanReg5 program ^20^, designed for the RPC by the IARC, was used to systematize the information of the incident cases, which allows eliminating duplicates and identifying multiple primary tumors. Additionally, the data is also validated with the IARC Tools and Registry Plus™ Link Plus programs ^20^ ^,^ ^21^.

For the estimation of the incidence rates, the population of the AMB of the study period, projected by DANE, was used as the denominator. Crude and standardized rates were calculated by direct method using the global standard population proposed by Segi and corrected by Doll ^22^. The analyses were performed in Stata 14 ^23^ and the program CanReg5 ^©^, version 5.00.42, software created by the International Agency for Research on Cancer (IARC), in collaboration with the International Association of Cancer Registries (IACR), available for free all members of the IACR ^24^.

## Results

During the 2008-2012 quinquennium, there were 8,775 incident cases of cancer in AMB (excluding non-melanoma skin cancer); (57.2%) occurred in women, (84.2%) were verified by microscopy (cytology, hematology or pathology), (7.6%) were detected only by death certificate, and (7.2%) by clinical history (Table 1). The percentage of cases identified by microscopy was higher in women than in men (88.0 and 81.4 respectively p <0.0001); in contrast, the percentage of cases identified by death certificate was higher in men than in women (9.1 and 6.5 *p* <0.0001).

**Table 1:** Quality index by cancer location and sex. Metropolitan Area of ​​Bucaramanga, 2008-2012.

Place	Men			Women			CIE-10
	n	% DCO	% MV	n	% DCO	% MV	
Oral cavity and pharynx	116	5.2	91.4	69	1.4	88.4	C00-C14
Esophagus	43	16.3	79.1	29	20.7	72.4	C15
Stomach	427	6.6	89.5	345	11.3	82.6	C16
Colorectal	353	5.9	91.0	457	4.8	92.3	C18-C21
Liver	90	35.6	46.7	69	31.9	44.9	C22
Pancreas	54	33.3	37.0	61	31.1	37.7	C25
Lung	251	21.9	66.1	200	23.5	64.0	C33-C34
Melanoma, skin	59	0.0	100.0	53	3.8	92.5	C43
Breast	12	8.3	83.3	1309	1.4	95.3	C50
Cervix	0.0	-	-	418	3.8	91.6	C53
Others in uterus	0.0	-	-	222	4.1	94.1	C54-C55
Ovary	0.0	-	-	215	8.4	85.1	C56
Prostate	980	7.3	76.8	0.0	-	-	C61
Urinary Bladder	83	3.6	95.2	39	5.1	87.2	C67
Thyroid	66	0.0	93.9	456	1.5	97.4	C73
Lymphoma	231	1.3	96.1	225	0.9	97.3	C81-C90;C96
Leukemia	180	1.7	96.1	151	2.6	96.7	C91-C95
Others	795	12.7	75.0	672	15.5	70.9	
All locations without skin	3,754	9.1	88.0	5,021	6.5	81.4	

DCO: death certificate as the only evidence

MV: microscopic verification

The average age at diagnosis was 57.3 years in women and 61.8 years in men (*p* <0.0001). The location of the most frequent malignant tumors in women were breast (26.1%), colorectal (9.1%), thyroid (9.1%), cervix (8.3%) and stomach (6.9%). In men, the most frequent cancers were: prostate (26.1%), stomach (11.4%), colorectal (9.4%), trachea bronchi and lung (6.7%), lymphoma and myeloma (6.2%) (Table 2). 

**Table 2:** Cases and crude and standardized incidence rates per 100,000 persons-year due to cancer and sex. Metropolitan Area of Bucaramanga, 2008-2012

Place	Both				Men				Women				CIE-10
	n	%	TC	ASR	n	%	TC	ASR	n	%	TC	ASR	
Oral cavity and Pharynx	185	2.1	3.4	4.3	116	3.1	4.5	4.6	69	1.4	2.4	2.1	C00-C14
Esophagus	72	0.8	1.3	1.2	43	1.1	1.7	1.7	29	0.6	1.0	0.8	C15
Stomach	772	8.8	14.3	13.1	427	11.4	16.5	17.1	345	6.9	12.3	10.2	C16
Colorectal	810	9.2	15	13.9	353	9.4	13.6	14.3	457	9.1	16.3	13.7	C18-C20
Liver	159	1.8	2.9	2.7	90	2.4	3.5	3.7	69	1.4	2.4	2.0	C22
Pancreas	115	1.3	2.1	2.0	54	1.4	2.1	2.2	61	1.2	2.2	1.8	C25
Lung	451	5.1	8.4	7.8	251	6.7	9.7	10.3	200	4.0	7.2	5.9	C33-C34
Melanoma, skin	112	1.3	2.1	2.0	59	1.6	2.3	2.3	53	1.1	1.9	1.7	C43
Breast	1.321	15.1	24.3	23.0	12	0.3	0.5	0.5	1,309	26.1	46.3	41.2	C50
Cervix	418	4.8	7.8	7.1	0	0.0	0.0	0.0	418	8.3	15.0	13.0	C53
Others in uterus	222	2.5	4.1	3.9	0	0.0	0.0	0.0	222	4.4	7.8	7.1	C54-C55
Ovary	215	2.5	4.0	3.8	0	0.0	0.0	0.5	215	4.3	7.7	7.0	C56
Prostate	980	11.2	18.1	17.3	980	26.1	37.7	40.9	0	0.0	0.0	0.0	C61
Urinary Bladder	122	1.4	2.3	2.0	83	2.2	3.2	3.3	39	0.8	1.4	1.1	C67
Thyroid	522	5.9	9.7	8.9	66	1.8	2.5	2.4	456	9.1	16.3	14.5	C73
Lymphoma	456	5.2	8.4	8.1	231	6.2	8.9	9.1	225	4.5	8.0	7.3	C81-C90;C96
Leukemia	331	3.8	6.1	6.3	180	4.8	6.9	7.3	151	3.0	5.4	5.5	C91-C95
Other neoplasms	1,467	16.7	27.1	25.9	795	21.2	30.5	31.6	672	13.4	23.9	21.3	
Total Cases without C44	8,775	100.0	162.2	153.1	3,754	100.0	144.5	151.7	5,021	100.0	178.7	157.2	

CR: Crude rates per 100,000

ASR: Standardized rates by age per 100,000

Rates are expressed per 100,000 persons-years. The cancer incidence rate standardized by age was 151.7 in men, and 157.2 in women. The standardized rates of the five most frequent cancers in men were prostate (40.9), stomach (17.1), colorectal (14.3), lung (10.3) and lymphoma (9.1). In women, they were breast (41.2), thyroid (14.5), colorectal (13.7), cervix (13.0) and stomach (10.2).

In women, the five tumor sites with a percentage of microscopic diagnosis greater than 95.2% were: thyroid, lymphoma, leukemia, kidney and breast. In men, they were skin melanoma, leukemia, lymphoma, bladder and thyroid. The type of cancer with the highest percentage of diagnosis by death certificate was: liver (31.9% in women and 35.6% in men), pancreas (31.1% in women and 33.3% in men) and lung (23.5% in women and 21.9% in men).

**Figure 1:**
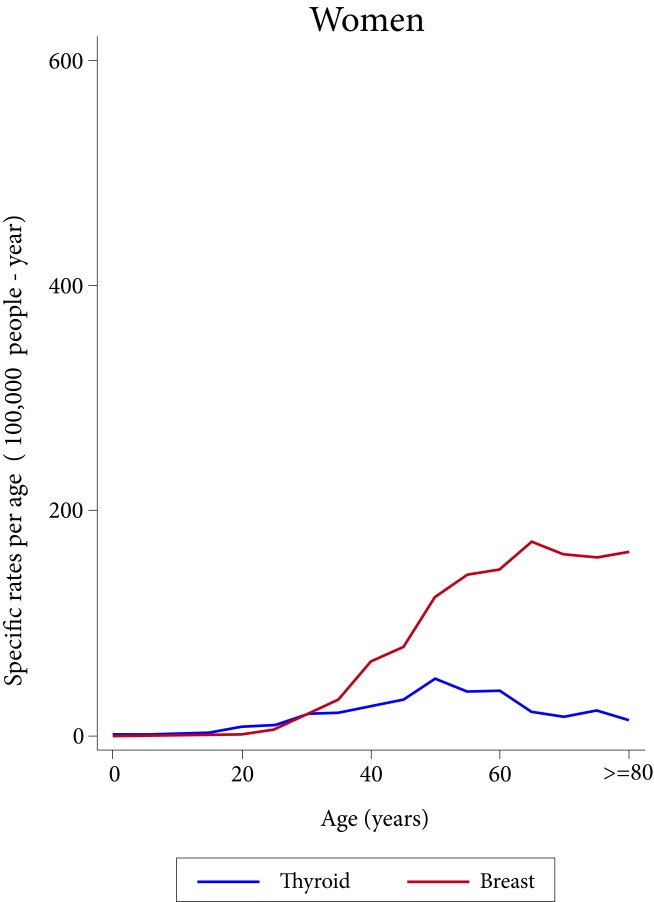
Age-specific incidence rates of the most frequent cancers in women in the metropolitan area of Bucaramanga, stratified by quinquennial 2008-2012.

**Figure 2:**
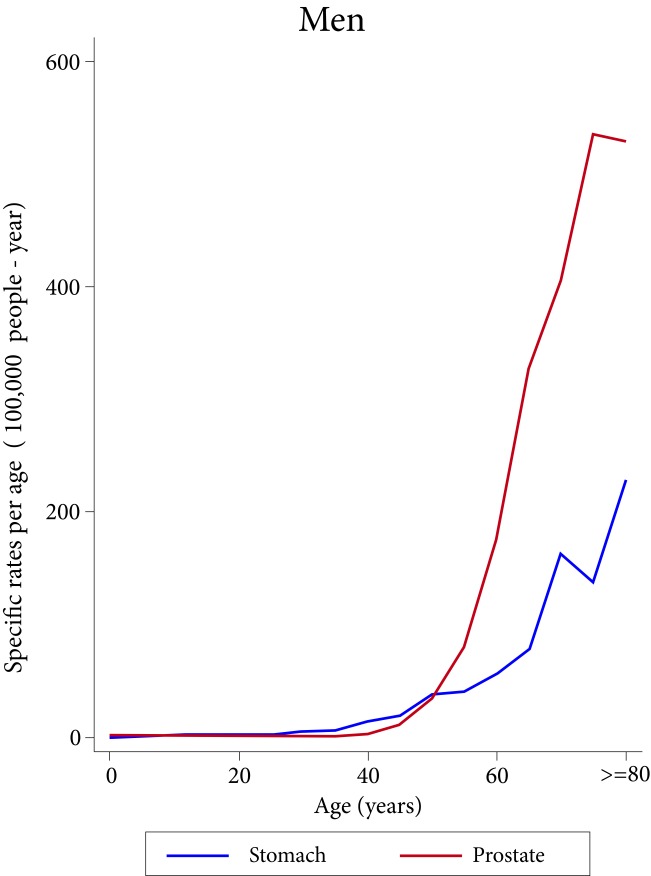
Age-specific incidence rates of the most frequent cancers in men in the metropolitan area of Bucaramanga, stratified by quinquennial 2008-2012.

The behavior of the age-specific incidence of the two most frequent types of cancer in women is different. Breast cancer rises sharply since the end of the third decade of life, reaching its peak at around 70 years of age; in contrast, the incidence of thyroid cancer begins in the middle of the second decade of life, and reaches its peak at around 55 years of age, with a magnitude 60% lower than that of breast cancer at this age (Fig. 1). In men, the incidence of gastric cancer increases after 30 years of age, and that of the prostate increases after 40 years of age, but the latter presents a much steeper upward curve, almost tripling the incidence of gastric cancer at 80 years of age (Fig. 2). 

During the 2008-2012 quinquennium, of 24,860 deaths registered in AMB, 4,998 (20.1%) corresponded to malignant neoplasms; 50.2% occurred in women. For the general population, the mortality rate standardized by cancer was 84.6 per 100,000 persons-years. The most frequent cancers were: stomach (13.9%), lung (11.4%), colorectal (9.0%), breast (7.1%), prostate (6.0%), leukemia (5.3%), liver (5.1%), lymphoma (5%), cervix (3.8%) and pancreas (3.5%).

Women had a standardized mortality rate by age of 78.0 per 100,000 women-year. The cancers that caused the greatest number of deaths were breast (13.3%), stomach (11.8%), colorectal (9.4%), lung (8.7%), cervix (7.1%), liver (4.8%), leukemia and lymphomas. (4.7% each). In men, the mortality rate standardized by age was 94.8 per 100,000 men-year, cancers of stomach (15.9%), lung (14.1%) and prostate (12.4%), colorectal (8.0%), liver (5.4%), leukemia (6.0%) and lymphoma (5.4%) (Table 3 ). 

**Table 3:** Cases and crude and standardized death rates (world SEGI population) per 100,000 persons-year, by location and sex. Metropolitan Area of Bucaramanga, 2008-2012

Place	Both				Men				Women			ICD-10
	n	%	CR	ASRA	n	%	CR	ASR	n	%	CR		ASR
Oral cavity and Pharynx	112	2.2	2.1	1.9	81	3.4	3.1	3.3	31	1.2	1.1	0.9	C00-C14
Esophagus	94	1.9	1.7	1.5	60	2.5	2.3	2.3	34	1.3	1.2	1.0	C15
Stomach	686	13.7	12.8	11.6	379	15.9	14.7	15.2	307	11.8	11	9.0	C16
Colorectal	455	9.0	8.3	7.4	204	8.0	7.9	8.1	241	9.4	8.6	6.7	C18-C20
Liver	254	5.1	4.7	4.4	130	5.4	5.0	5.3	124	4.8	4.4	3.7	C22
Pancreas	175	3.5	3.3	2.9	80	3.3	3.1	3.2	95	3.6	3.4	2.7	C25
Lung	563	11.3	10.5	9.6	337	14.1	13.0	13.7	226	8.7	8.1	6.6	C33-C34
Melanoma, skin	50	1.0	0.9	0.8	26	1.1	1.0	1.0	24	0.9	0.8	0.7	C43
Breast	349	7.0	6.5	6.1	3	0.1	0.1	0.1	346	13.3	12.4	10.8	C50
Cervix	186	3.7	3.5	3.1					186	7.1	6.7	5.7	C53
Others in uterus	55	1.1	1.0	0.9					55	2.1	2.0	1.6	C54-C55
Ovary	128	2.6	2.4	2.2					128	4.9	4.6	4.0	C56
Prostate	297	5.9	5.5	4.6	297	12.4	11.5	11.3					C61
Urinary Bladder	63	1.3	1.2	1.0	36	1.5	1.4	1.4	27	1.0	1.0	0.7	C67
Lymphoma	251	5.0	4.7	4.3	129	5.4	5.0	5.1	122	4.7	4.4	3.7	C81-C90;C96
Leukemia	266	5.3	4.9	4.7	143	6.0	5.5	5.6	123	4.7	4.4	4.1	C91-C95
Other locations	1.013	20.3	18.8	17.3	480	20.1	18.5	19.1	533	20.4	19.1	16.0	
Total cases of cancer	4,997	100.0	92.9	84.6	2,390	100.0	92.4	94.8	2,607	100.0	93.3	78.0	

CR: Crude rates per 100,000

ASR: Standardized rates by age per 100,000

## Discussion

This study presents the results of cancer incidence and mortality in the quinquennium 2008-2012, thus providing continuity with the data for the 2003-2007 quinquennium ^25^. In general, the quality of the data analyzed was better than for the previous quinquennium, because the percentage of cases that were detected only by death certificate decreased.

In the interpretation of data, it is important to bear in mind that the classification of the cases used in the current study was based on the first revision of the ICD-O-3, which includes changes in the codes related to the behavior of some tumor lesions and morphological codes of hematolymphoid and central nervous system neoplasms, which were not taken into account in the analysis of the previous quinquennium.

When compared with the 2003-2007 quinquennium, the average age at diagnosis moment was similar for both sexes. The total number of new cases (excluding non-melanoma skin cancer) increased 6.7%, as well as the percentage representation of women, which went from 54.3% to 57.2%. However, the overall incidence rate in men and women decreased, especially in men, because the male/female ratio (m/f) was 1.0, showing a behavior contrary to that reported by the Population Registry of Cali Cancer (RPCC, for its initials in Spanish) for the same period (ratio m/f of 1.1) ^26^, but similar to the behavior reported for previous years in countries such as Ecuador, Peru and Mexico ^27^.

The standardized rates of incidence in the 2008-2012 period in AMB were lower than those reported by RPCC in the same period ^26^, as well as those of the South American population (206.7 per 100,000 men-year and 180.6 per 100,000 women -year) ^27^ and worldwide (182.0 per 100,000 men-year and 165.2 per 100,000 women-year) ^26^.

Breast cancer was the most frequent in women from AMB, with percentage representation and standardized incidence rate similar to those of the previous quinquennium (41.2 versus 41.9 per 100,000 women-year) ^25^, and the global behavior reported for the year 2012 ^26^. However, the order of the other more frequent cancers in women from AMB was modified, placing thyroid cancer in the second place, followed by colorectal cancer, and displacing cervix cancer to the fourth place; cervix cancer had occupied the second place in the previous quinquennium, showing a sustained tendency to decrease in its rate since 2000. Stomach cancer was the fifth cancer in frequency, with the same incidence as in the previous quinquennium.

According to the estimates of the National Health Observatory (ONS, for its initials in Spanish) in Colombia for the year 2012, breast cancer, followed by cervical cancer, continued to have the highest incidence in women, with magnitudes higher than those of AMB (47.3 and 16.3 per 100,000 women-year, respectively); while cancer of the colon, rectum and anus (7.8 per 100,000 women-year), thyroid cancer (11.2 per 100,000 women-year) and stomach cancer (9.6 per 100,000 women-year) had lower rates. Particularly, the latter showed a marked downward trend in the country during the 2010-2014 period ^28^, in contrast to the stability of its behavior in women from AMB. 

In men, the order of the five cancers with the highest incidence remains the same from one quinquennium to another; all but colorectal cancer showed a decrease in the standardized incidence rate compared to the quinquennium 2003-2007 ^25^. These five types of cancer were also the ones with the highest incidence for men in the country in 2012, although with standardized rates higher than those of AMB and with an increasing tendency in the quinquennium 2010-2014; with the exception of stomach cancer, which showed a clear tendency to decrease at national level ^28^.

In relation to mortality, the profile of AMB is similar to the quinquennium 2003-2007, although with lower rates in most locations; especially stomach cancer, which decreased particularly in men, from 20.1 to 15.2 per 100,000 men- year. In contrast, the death rate for colorectal cancer increased in men, as did the mortality rate from leukemia, which increased in men and women, with mortality in the period 2008-2012 being 2.4 times higher than in the previous quinquennium ^25^. 

The outlook in AMB contrasts with that of the country, which according to ONS calculations had higher standardized mortality rates and a tendency to increase in several locations. However, mortality from leukemia showed an opposite behavior compared to the national estimate for 2012, with AMB reaching a 46.5% higher rate in men and 70.5% higher in women in the 2008-2012 quinquennium. Unlike the behavior of the country, whose mortality from cancer of the colon, rectum and anus increased in men and women during the quinquennium 2010-2014, in AMB this behavior was only observed in men ^28^.

The behavior of the country reflects the double burden of cancer faced by the Central and South America regions, which manifests in high rates of cancer related to infection (cervix, stomach and liver) and an increase in cancers related to lifestyle (prostate, breast, colon and rectum), the latter ones possibly have to do with aspects of economic development, such as the increase in the age of first pregnancy, lower parity, smoking and alcohol consumption, diets poor in fruits and vegetables , obesity and physical inactivity ^27^.

Although in several locations of tumors, AMB has lower incidences compared to the total population of the country, here the double burden is also appreciated. In addition, the magnitude of colorectal cancer and its tendency to increase in both sexes is noteworthy, as well as the high frequency of stomach cancer and the increased incidence of thyroid cancer in women. Also, although the risk of dying from cancer is lower in AMB than in the rest of the country for almost all locations, and it was lower than in the previous quinquennium, the increase in mortality from colon cancer and rectum in men, and from leukemia for both sexes is significant.

This profile raises more attention to the prevention of colon and rectum cancer in AMB, where the magnitude of the incidence is similar for both sexes, unlike what happens in much of the world. This type of cancer shares with breast cancer several prevention measures, such as the maintenance of body weight, increased physical activity and decreased consumption of alcohol, red and processed meats, and tobacco ^1^.

It should be noted that the prevalence of overweight or obesity in the province of Santander is 50.1%, higher in men, although abdominal obesity is higher in women (46.6% versus 40.11%), the consumption of fruits and vegetables is very low (94.9% did not reach the recommended daily consumption) ^29^, and gender and socioeconomic inequalities have been documented in the practice of physical activity, with women being less active, especially those engaged in unpaid work ^30^. Inequalities in lifestyles demand comprehensive approaches that deepen the role of social structures in the configuration of health decisions and practices, as these do not depend solely on personal decisions ^31^ ^,^ ^32^. 

Improving and promoting equity at all preventive levels in relation to stomach cancer is also a priority for AMB, especially when marked socio-economic inequalities have been documented in the survival of patients, despite the existence of the "universal" system of social security in health ^33^.

With regard to thyroid cancer, this presents an increase on incidence rates similar to the data reported for several regions of the world and the country. In some countries such as the United States, the increase in incidence rates was recorded three decades ago due to a greater use of diagnostic methods such as ultrasound, which allowed them to find very small nodules that would probably go unnoticed for a long time; that is, the relative increase in incidence could be influenced by the diagnosis.

Finally, the behavior of hematolymphoid neoplasms, especially in leukemia, with mortality rates that have doubled the rates of the previous quinquennium in our region, raises concerns about the factors that have impacted this marked increase in mortality rates. It is important to explore modifiable factors, especially those that have been associated with barriers in the health care of patients with cancer, such as administrative, economic and cultural factors, with delays in the opportunity for diagnosis and treatment ^34^. 

## Conclusions

This study presents the magnitude of cancer in the Metropolitan Area of ​​Bucaramanga during the quinquennium 2008-2012, comparatively with the quinquennium 2003-2007. Positive behaviors are revealed for several cancers, which show a decrease in the incidence of one quinquennium to another, or magnitudes lower than those of the country; however, the magnitude and tendency to increase in colorectal cancer for both sexes is to significant, as well as the high frequency of stomach cancer, and the increased incidence of thyroid cancer in women. Likewise, mortality from colon and rectal cancer in men, and from leukemia for both sexes has increased, which requires further investigation and the strengthening of preventive measures.
