# Supplementary material for: Cancer incidence and mortality in Bucaramanga, Colombia. 2008-2012
Source: Colomb Med (Cali). 2018 Mar 30;49(1):73–80. doi: 10.25100/cm.v49i1.3632 (PMC6018816; doi:10.25100/cm.v49i1.3632)
Supplement: Related texts

# Research article: Incidencia y mortalidad por cáncer en Bucaramanga, Colombia. 2008-2012

## Introducción 

El cáncer constituye una enorme carga en el mundo, ocasionando cerca de 14.1 millones de casos nuevos y 8.2 millones de muertes en el 2012. La carga por cáncer es cada vez mayor en los países menos desarrollados, de donde provienen alrededor del 57% de los casos nuevos y el 65% de las muertes ocasionadas por esta enfermedad. Se estima que el 48% de los casos prevalentes de cáncer a cinco años del diagnóstico se presentaron en las regiones con menor desarrollo económico ^1^^,^^2^.

La incidencia del cáncer está aumentando. Se espera que para el año 2025 se presenten más de 20 millones de casos nuevos, y una mayor carga en los países de bajos y medianos ingresos ^3^^,^^4^. Esta tendencia se atribuye al crecimiento y envejecimiento de la población, así como a una creciente prevalencia de factores de riesgo claramente establecidos como el tabaquismo, el sobrepeso, la inactividad física y los cambios en los patrones reproductivos asociados con la urbanización y el desarrollo económico ^1^^,^^2^. 

Para impactar en la reducción de la problemática generada por esta enfermedad, la OMS promueve la creación de programas efectivos que logren mejorar la detección precoz, el diagnóstico oportuno y un tratamiento específico y efectivo ^5^.

Asimismo, lidera programas de control del cáncer con la promoción de políticas, planes y programas nacionales de control del cáncer. Una de las estrategias ha sido la promoción, el establecimiento y fortalecimiento del monitoreo y la evaluación de esta enfermedad por medio de registros, así como de investigaciones dirigidos a la carga de la enfermedad y mayor disponibilidad de recursos para su atención ^6^.

Los Registros Poblacionales de Cáncer (RPCs) son sistemas de información imprescindibles para los programas del control de cáncer; estos permiten determinar frecuencias, incidencias, mortalidad y tendencias, como resultado de actividades encaminadas a recoger, analizar y difundir de manera continua, información de los casos de cáncer que ocurren en una población específica, con garantía de la calidad de los datos (exhaustividad, precisión, y comparabilidad) ^7^^,^^8^.

En Colombia existen cuatro RPCs con reconocimiento internacional. Uno de estos, es el Registro Poblacional de Cáncer del Área Metropolitana de Bucaramanga (RPC-AMB) que ha logrado consolidar un trabajo continuo durante 18 años de funcionamiento ^9^^,^^10^.

Con el liderazgo del Ministerio de Salud y de la Protección social se organiza el Sistema Nacional de Información en Cáncer y se crea el Observatorio Nacional de Cáncer para Colombia liderado por el Instituto Nacional de Cancerología de Colombia (INC) con el fin de conocer el perfil territorial y la evaluación del impacto de los programas de control de cáncer ^11^. Los RPCs son una pieza fundamental de esta iniciativa ^12^ y a través de ellos se puede conocer la epidemiología de la enfermedad con lo cual se avanza en el cumplimiento de uno de los objetivos pilares del Plan Decenal de Control de Cáncer ^13^. 

En este estudio se describen las tasas de incidencia y mortalidad totales y específicas por sexo, correspondientes al Área Metropolitana de Bucaramanga en el periodo 2008-2012.

## Materiales y Métodos

Se planteó un estudio poblacional descriptivo, con los casos incidentes de cáncer invasivos registrados en las bases de datos del RPC-AMB en el quinquenio 2008-2012. 

### Área geográfica

El AMB es una zona geográfica conformada por cuatro municipios del departamento de Santander: Bucaramanga (capital), Floridablanca, Girón y Piedecuesta. Está ubicada en la región andina y cuenta con una extensión de 1,479 Km^2^. Cada municipio es independiente en su organización política, pero están completamente relacionados desde la perspectiva económica y social ^14^.

Según proyecciones de población del Departamento Administrativo Nacional de Estadística (DANE), a mitad del periodo 2008-2012 el AMB contaba con 1,074,000 (53.5% del total departamental), 94.3% eran residentes urbanos, 51.9% eran mujeres, 24.5% menores de 15 años y 20.4% mayores de 50 años ^15^.

El AMB es un importante centro de referencia para la atención de pacientes con cáncer en la zona nororiental del país, cuenta con instituciones públicas (hospitales) y privadas (clínicas, centros médicos especializados, centros de diagnóstico, centros oncológicos y médicos especialistas en todas las áreas) que proveen diversos servicios oncológicos para el diagnóstico y tratamiento.

### Registro Poblacional de Cáncer del Área Metropolitana de Bucaramanga

El RPC-AMB ha realizado actividades permanentes desde abril del 2000, fruto del compromiso asumido por la UNAB. Los recursos económicos son asignados en las convocatorias de investigación de la Universidad y recibe cofinanciación anual del Instituto Nacional de Cancerología.

### Método de recolección y procesamiento de los datos

El RPC-AMB hace recolección activa de los casos incidentes mediante visitas periódicas a las fuentes de información: laboratorios de patología y hematología, egresos hospitalarios, centros de imagenología, centros de tamizaje, voluntariados, centros de oncología, médicos especialistas, autopsias y certificados de defunción que son obtenidos en la Secretaría de Salud Departamental.

Un caso registrable es toda neoplasia maligna de cualquier localización incluyendo las neoplasias benignas del Sistema Nervioso Central (SNC) y los carcinomas *in situ* que hayan sido diagnosticadas después del 1^ro^ de enero del año 2000 en residentes del AMB, tanto de zona urbana como rural, independiente del método diagnóstico utilizado, de tal forma que también se incluyen los casos identificados por certificado de defunción. Se excluye el cáncer de piel no melanoma (basocelular y escamocelular).

Los casos obtenidos por egreso hospitalario y certificados de defunción siguen un proceso de verificación del diagnóstico, mediante la búsqueda de la historia clínica. Cuando no se obtiene información adicional del caso obtenido por el certificado de defunción, se registra como caso identificado únicamente por certificado de defunción (DCO por su sigla en inglés). 

La codificación de los casos de cáncer es realizada con supervisión de la Directora del RPC, por personal entrenado en la Clasificación Internacional de Enfermedades para Oncología (CIE-O) - 3ª edición, 1ª revisión, publicado por la Organización Mundial de la Salud (OMS) en el 2013, y en los criterios de la IARC para los tumores primarios múltiples ^16^^,^^17^.

El RPC-AMB cumple con normas de confidencialidad siguiendo parámetros de la IARC y los compromisos de ética de la investigación. Sólo el personal del RPC-AMB accede a la información de cada caso que permite un trabajo exhaustivo en el control de la duplicidad de la información ^8^^,^^18^. 

La fuente de información de defunciones fue el DANE, entidad oficial que recoge, organiza y codifica la causa básica de mortalidad utilizando la décima revisión de la Clasificación Internacional de Enfermedades (CIE 10) ^19^.

El programa CanReg5 ^20^, diseñado para los RPC por la IARC, fue empleado para la sistematización de la información de los casos incidentes, el cual permite eliminar duplicados e identificar tumores primarios múltiples. Adicionalmente, los datos también se validan con los programas IARCTools y Registry Plus™ Link Plus ^20^^,^^21^.

Para la estimación de las tasas de incidencia se utilizó como denominador la población del AMB del período de estudio, proyectada por el DANE. Se calcularon tasas crudas y estandarizadas por método directo empleando la población estándar mundial propuesta por Segi y corregida por Doll ^22^. Los análisis se realizaron en Stata 14 ^23^ y el programa CanReg5^®^, versión 5.00.42, software creado por la International Agency for Research on Cancer (IARC) en colaboración con la International Association of Cancer Registries (IACR), disponible gratuitamente para todos los miembros de la IACR ^24^. 

## Resultados 

Durante el quinquenio 2008-2012 se registraron 8,775 casos incidentes de cáncer en el AMB (excluyendo cáncer de piel no melanoma), (57.2%) se presentaron en mujeres, (84.2%) fueron verificados por microscopía (citología, hematología o patología), (7.6%) fueron detectados únicamente por certificado de defunción y (7.2%) por historia clínica (Tabla 1). El porcentaje de casos identificados por microscopía fue mayor en mujeres que en hombres (88.0 y 81.4 respectivamente *p* <0.0001), en contraste, el porcentaje de casos identificados por certificado de defunción fue mayor en hombres que en mujeres (9.1 y 6.5 *p* <0.0001). 

**Tabla 1:** Indice de calidad por localización del cáncer y sexo. Área Metropolitana de Bucaramanga, 2008-2012.

Sitio	Hombres			Mujeres			CIE-10
	n	% DCO	% VM	n	% DCO	% VM	
Cavidad oral y faringe	116	5.2	91.4	69	1.4	88.4	C00-C14
Esófago	43	16.3	79.1	29	20.7	72.4	C15
Estómago	427	6.6	89.5	345	11.3	82.6	C16
Colorrecto	353	5.9	91.0	457	4.8	92.3	C18-C21
Hígado	90	35.6	46.7	69	31.9	44.9	C22
Páncreas	54	33.3	37.0	61	31.1	37.7	C25
Pulmón	251	21.9	66.1	200	23.5	64.0	C33-C34
Melanoma, piel	59	0.0	100.0	53	3.8	92.5	C43
Mama	12	8.3	83.3	1,309	1.4	95.3	C50
Cuello del útero	0	-	-	418	3.8	91.6	C53
Otros útero	0	-	-	222	4.1	94.1	C54-C55
Ovario	0	-	-	215	8.4	85.1	C56
Próstata	980	7.3	76.8	0	-	-	C61
Vejiga	83	3.6	95.2	39	5.1	87.2	C67
Tiroides	66	0.0	93.9	456	1.5	97.4	C73
Linfoma	231	1.3	96.1	225	0.9	97.3	C81-C90;C96
Leucemia	180	1.7	96.1	151	2.6	96.7	C91-C95
Otros	795	12.7	75.0	672	15.5	70.9	
Todas las localizaciones sin piel	3,754	9.1	88.0	5,021	6.5	81.4	

%DCO: Porcentaje de casos por certificado de defunción como única evidencia

%VM: Porcentaje de casos con verificación microscópica

El promedio de edad al momento del diagnóstico fue de 57.3 años en las mujeres y 61.8 años en los hombres (*p* <0.0001). La localización de los tumores malignos más frecuentes en las mujeres fueron mama (26.1%), colorrectal (9.1%), tiroides (9.1%), cuello de útero (8.3%) y estómago (6.9%). En los hombres, los tipos de cáncer más frecuentes fueron: próstata (26.1%), estómago (11.4%), colorrectal (9.4%), tráquea bronquios y pulmón (6.7%), linfomas y mielomas (6.2%) (Tabla 2).

**Tabla 2:** Casos y tasas de incidencia crudas y estandarizadas por 100,000 personas-año por cáncer y sexo. Área Metropolitana de Bucaramanga, 2008-2012

SITIO	Ambos				Hombres				Mujeres				CIE-10
	n	%	TC	TEE	n	%	TC	TEE	n	%	TC	TEE	
Cavidad Oral y Faringe	185	2.1	3.4	4.3	116	3.1	4.5	4.6	69	1.4	2.4	2.1	C00-C14
Esófago	72	0.8	1.3	1.2	43	1.1	1.7	1.7	29	0.6	1	0.8	C15
Estómago	772	8.8	14.3	13.1	427	11.4	16.5	17.1	345	6.9	12.3	10.2	C16
Colon, recto	810	9.2	15.0	13.9	353	9.4	13.6	14.3	457	9.1	16.3	13.7	C18-C20
Hígado	159	1.8	2.9	2.7	90	2.4	3.5	3.7	69	1.4	2.4	2.0	C22
Páncreas	115	1.3	2.1	2.0	54	1.4	2.1	2.2	61	1.2	2.2	1.8	C25
Pulmón	451	5.1	8.4	7.8	251	6.7	9.7	10.3	200	4.0	7.2	5.9	C33-C34
Melanoma, Piel	112	1.3	2.1	2.0	59	1.6	2.3	2.3	53	1.1	1.9	1.7	C43
Mama	1,321	15.1	24.3	23.0	12	0.3	0.5	0.5	1,309	26.1	46.3	41.2	C50
Cuello del útero	418	4.8	7.8	7.1	0	0.0	0.0	0.0	418	8.3	15.0	13.0	C53
Otros útero	222	2.5	4.1	3.9	0	0.0	0.0	0.0	222	4.4	7.8	7.1	C54-C55
Ovario	215	2.5	4.0	3.8	0	0.0	0.0	0.5	215	4.3	7.7	7.0	C56
Próstata	980	11.2	18.1	17.3	980	26.1	37.7	40.9	0	0.0	0.0	0.0	C61
Vejiga	122	1.4	2.3	2.0	83	2.2	3.2	3.3	39	0.8	1.4	1.1	C67
Tiroides	522	5.9	9.7	8.9	66	1.8	2.5	2.4	456	9.1	16.3	14.5	C73
Linfoma	456	5.2	8.4	8.1	231	6.2	8.9	9.1	225	4.5	8.0	7.3	C81-C90;C96
Leucemia	331	3.8	6.1	6.3	180	4.8	6.9	7.3	151	3.0	5.4	5.5	C91-C95
Otras Neo	1,467	16.7	27.1	25.9	795	21.2	30.5	31.6	672	13.4	23.9	21.3	
Total Casos sin C44	8,775	100.0	162.2	153.1	3,754	100	144.5	151.7	5,021	100	178.7	157.2	

TC: Tasas crudas por 100,000

TEE: Tasas estandarizadas por edad por 100,000

Las tasas se expresan por 100,000 personas-año. La tasa de incidencia de cáncer estandarizada por edad fue de 151.7 en hombres, y 157.2 en mujeres. Las tasas estandarizadas de los cinco tipos de cáncer más frecuentes en hombres fueron próstata (40.9), estómago (17.1), colorrectal (14.3), pulmón (10.3) y linfoma (9.1). En mujeres fueron mama (41.2), tiroides (14.5), colorrectal (13.7), cuello de útero (13.0) y estómago (10.2).

En mujeres las cinco localizaciones de tumores con porcentaje de diagnóstico microscópico mayor al 95.2% fueron: tiroides, linfomas, leucemias, riñón y mama. En hombres fueron melanoma de la piel, leucemia, linfoma, vejiga y tiroides. El tipo de cáncer con mayor porcentaje de diagnóstico por certificado de defunción fue: hígado (31.9% en mujeres y 35.6% en hombres), páncreas (31.1% en mujeres y 33.3% en hombres) y pulmón (23.5% en mujeres y 21.9% en hombres).

El comportamiento de la incidencia específica por edad de los dos tipos de cáncer más frecuentes en las mujeres fue diferente. El cáncer de mama asciende de manera acentuada desde el final de la tercera década de vida, alcanzando su pico máximo alrededor de los 70 años, mientras que la incidencia del cáncer de tiroides inicia a mediados de la segunda década de vida y alcanza su pico alrededor de los 55 años con una magnitud 60% inferior a la del cáncer de mama en esta edad (Fig. 1). En los hombres, la incidencia del cáncer gástrico aumenta después de los 30 años y el de próstata se incrementa a partir de los cuarenta años, pero este último presenta una curva ascendente mucho más pronunciada llegando casi a triplicar la incidencia del cáncer gástrico a los 80 años (Fig. 2).

**Figura 1:** Tasas de incidencia específicas por edad de los cánceres más frecuentes en mujeres en el área metropolitana de Bucaramanga estratificada por quinquenios 2008-2012.

**Figura 2:** Tasas de incidencia específicas por edad de los canceres más frecuentes en hombres en el área metropolitana de Bucaramanga estratificada por quinquenios 2008-2012

Durante el quinquenio 2008-2012, de las 24,860 muertes registradas en el AMB, 4,998 (20.1%) correspondieron a neoplasias malignas, 50.2% ocurrieron en mujeres. En la población general la tasa de mortalidad estandarizada por cáncer fue de 84.6 por 100,000 personas-año. Los tipos de cáncer con mayor frecuencia fueron: estómago (13.9%), pulmón (11.4%), colorrectal (9.0%), mama (7.1%), próstata (6.0%), leucemia (5.3%), hígado (5.1%), linfoma (5.0%), cuello de útero (3.8%) y páncreas (3.5%). 

Las mujeres tuvieron una tasa de mortalidad estandarizada por edad de 78.0 por 100,000 mujeres-año. Los cánceres que mayor número de muertes ocasionaron fueron mama (13.3%), estómago (11.8%), colorrectal (9.4%), pulmón (8.7%), cuello de útero (7.1%), hígado (4.8%), leucemias y linfomas (4.7% cada uno). En hombres, la tasa de mortalidad estandarizada por edad fue de 94.8 por 100,000 hombres-año, los cánceres de estómago (15.9%), pulmón (14.1%) y próstata (12.4%), colorrectal (8.0%), hígado (5.4%), leucemia (6.0%) y linfoma (5.4%) (Tabla 3). 

**Tabla 3:** Casos y tasas de mortalidad crudas y estandarizadas (población mundial Segi) por 100,000 personas-año, por localización y sexo. Área Metropolitana de Bucaramanga, 2008-2012

SITIO	Ambos			Hombres				Mujeres				ICD-10
	n	%	TC	TEE	n	%	TC	TEE	n	%	TC		TEE
Cavidad oral y faringe	112	2.2	2.1	1.9	81	3.4	3.1	3.3	31	1.2	1.1	0.9	C00-C14
Esófago	94	1.9	1.7	1.5	60	2.5	2.3	2.3	34	1.3	1.2	1.0	C15
Estómago	686	13.7	12.8	11.6	379	15.9	14.7	15.2	307	11.8	11.0	9.0	C16
Colon, recto	455	9.0	8.3	7.4	204	8.0	7.9	8.1	241	9.4	8.6	6.7	C18-C20
Hígado	254	5.1	4.7	4.4	130	5.4	5.0	5.3	124	4.8	4.4	3.7	C22
Páncreas	175	3.5	3.3	2.9	80	3.3	3.1	3.2	95	3.6	3.4	2.7	C25
Pulmón	563	11.3	10.5	9.6	337	14.1	13.0	13.7	226	8.7	8.1	6.6	C33-C34
Melanoma de piel	50	1.0	0.9	0.8	26	1.1	1.0	1.0	24	0.9	0.8	0.7	C43
Mama	349	7.0	6.5	6.1	3	0.1	0.1	0.1	346	13.3	12.4	10.8	C50
Cuello de útero	186	3.7	3.5	3.1					186	7.1	6.7	5.7	C53
Otros útero	55	1.1	1.0	0.9					55	2.1	2.0	1.6	C54-C55
Ovario	128	2.6	2.4	2.2					128	4.9	4.6	4.0	C56
Próstata	297	5.9	5.5	4.6	297	12.4	11.5	11.3					C61
Vejiga	63	1.3	1.2	1.0	36	1.5	1.4	1.4	27	1.0	1.0	0.7	C67
Linfoma	251	5.0	4.7	4.3	129	5.4	5.0	5.1	122	4.7	4.4	3.7	C81-C90;C96
Leucemia	266	5.3	4.9	4.7	143	6.0	5.5	5.6	123	4.7	4.4	4.1	C91-C95
Otras localizaciones	1.013	20.3	18.8	17.3	480	20.1	18.5	19.1	533	20.4	19.1	16.0	
Total casos cáncer	4,997	100.0	92.9	84.6	2,390	100.0	92.4	94.8	2,607	100.0	93.3	78.0	

TC: Tasas crudas por 100,000

TEE: Tasas estandarizadas por edad por 100,000

## Discusión

Este estudio presenta los resultados de la incidencia y la mortalidad por cáncer en el quinquenio 2008-2012, brindando así continuidad con los datos del quinquenio 2003-2007 ^25^. En general la calidad de los datos analizados fue mejor que el quinquenio anterior, porque disminuyó el porcentaje de casos que fueron detectados únicamente por certificado de defunción. 

En la interpretación de los datos es importante tener en cuenta que la clasificación de los casos empleada en el actual estudio se basó en la primera revisión del CIE-O-3, la cual incluye cambios en los códigos relacionados con el comportamiento de algunas lesiones tumorales y códigos morfológicos de neoplasias hematolinfoides y del sistema nervioso central; los cuales no se tuvieron en cuenta en el análisis del quinquenio anterior.

Al comparar con el quinquenio 2003-2007, la edad promedio al momento del diagnóstico en los casos de ambos sexos fue similar. El número total de casos nuevos (excluyendo cáncer de piel no melanoma) aumentó 6.7%, así como la representación porcentual de las mujeres, que pasó de 54.3% a 57.2%. No obstante, la tasa de incidencia global en hombres y mujeres disminuyó, especialmente en hombres, por cuanto la relación hombre/mujer (h/m) fue de 1.0, mostrando un comportamiento contrario a lo reportado por el Registro Poblacional de Cáncer de Cali (RPCC) para el mismo periodo (razón h/m de 1.1) ^26^, pero similar al comportamiento reportado desde años anteriores en países como Ecuador, Perú y México ^27^. 

Las tasas estandarizadas de incidencia en el quinquenio 2008-2012 en el AMB fueron inferiores a las reportadas por el RPCC en el mismo periodo ^26^, así como a las de la población Suramericana (206.7 por 100,000 hombres-año y 180.6 por 100,000 mujeres-año) ^27^ y mundial (182.0 por 100,000 hombres-año y 165.2 por 100,000 mujeres-año) ^26^. 

El cáncer de mama fue el más frecuente en mujeres del AMB, con representación porcentual y tasa de incidencia estandarizada similares a las del quinquenio anterior (41.2 versus 41.9 por 100,000 mujeres-año) ^25^ y al comportamiento mundial reportado para el año 2012 ^26^. No obstante, el orden de los demás tipos de cáncer con mayor frecuencia en las mujeres del AMB se modificó, ubicándose el cáncer de tiroides en el segundo lugar seguido por el cáncer colorrectal, desplazando al cáncer de cuello de útero al cuarto lugar, el cual había ocupado el segundo lugar en el quinquenio anterior, mostrando así una tendencia sostenida a la disminución en su tasa desde el año 2000. El cáncer de estómago fue el quinto cáncer en frecuencia, con igual incidencia a la del quinquenio anterior. 

De acuerdo con las estimaciones del Observatorio Nacional de Salud (ONS) en Colombia para el año 2012, el cáncer de mama seguido del cáncer de cuello uterino continúan siendo los de mayor incidencia en mujeres, con magnitudes superiores a las del AMB (47.3 y 16.3 por 100,000 mujeres-año, respectivamente), mientras que el cáncer de colon, recto y ano (7.8 por 100,000 mujeres-año), de tiroides (11.2 por 100.000 mujeres-año) y de estómago (9.6 por 100,000 mujeres-año) cuentan con tasas inferiores. Particularmente este último mostró una marcada tendencia a la disminución en el país durante el periodo 2010-2014 ^28^, en contraste con la estabilidad de su comportamiento en las mujeres del AMB. 

En hombres, el orden de los cinco cánceres con mayor incidencia se mantuvo de un quinquenio a otro; todos a excepción del cáncer colorrectal mostraron una disminución en la tasa de incidencia estandarizada en comparación con el quinquenio 2003-2007 ^25^. Estos cinco tipos de cáncer también son los de mayor incidencia en los hombres del país en el año 2012, aunque con tasas estandarizadas mayores a las del AMB y con tendencia al aumento en el quinquenio 2010-2014, a excepción del cáncer de estómago, que presentó una clara tendencia a la disminución en el ámbito nacional ^28^.

En relación con la mortalidad, el perfil del AMB es similar al quinquenio 2003-2007, aunque con tasas más bajas en la mayoría de localizaciones, especialmente por cáncer de estómago que disminuyó particularmente en hombres, pasando de (20.1 a 15.2 por 100,000 hombres-año). En contraste, la tasa de mortalidad por cáncer de colorrectal aumentó en hombres así como la tasa de mortalidad por leucemia, que aumentó en hombres y en mujeres, siendo la mortalidad en el periodo 2008-2012 de 2.4 veces mayor a la del quinquenio anterior ^25^.

El panorama en el AMB contrasta con el del país, que según cálculos del ONS contó con tasas estandarizadas de mortalidad más altas y con tendencia al aumento en varias localizaciones. Sin embargo, en la mortalidad por leucemia se observó un comportamiento contrario en comparación con el estimado nacional a 2012, alcanzando el AMB una tasa 46.5% superior en los hombres y 70.5% superior en las mujeres en el quinquenio 2008-2012. A diferencia del comportamiento del país, cuya mortalidad por cáncer de colon, recto y ano aumentó en hombres como en mujeres durante el quinquenio 2010-2014, en el AMB este comportamiento solo se observó en hombres ^28^.

El comportamiento del país refleja la doble carga por cáncer que enfrenta la región de Centro y Sur América, que se manifiesta en altas tasas de cáncer relacionadas con infección (cuello de útero, estómago e hígado) y un incremento en cánceres relacionados con el estilo de vida (próstata, mama y colon y recto ), estos últimos posiblemente tienen que ver con aspectos del desarrollo económico, tales como el aumento en la edad del primer embarazo, menor paridad, consumo de tabaco y alcohol, y dietas pobres en frutas y vegetales, obesidad e inactividad física ^27^. 

Aunque en varias localizaciones de tumores, el AMB cuenta con incidencias más bajas en comparación con la población total del país, en este también se aprecia la doble carga; y es de resaltar la magnitud y la tendencia al aumento del cáncer de colorrectal en ambos sexos, así como la alta frecuencia del cáncer de estómago y el aumento de la incidencia por cáncer de tiroides en mujeres. Asimismo, aunque el riesgo de morir por cáncer es más bajo en el AMB que en la población nacional en casi todas las localizaciones, y fue inferior al del AMB en el quinquenio anterior, es de resaltar el aumento en la mortalidad por cáncer de colon y recto en hombres y por leucemias en ambos sexos. 

Este perfil plantea una mayor atención a la prevención del cáncer de colon y recto en el AMB, en donde la magnitud de la incidencia es similar en ambos sexos, a diferencia de lo que ocurre en gran parte del mundo. Este tipo de cáncer comparte con el de mama varias medidas de prevención, tales como el mantenimiento del peso corporal, aumento de la actividad física y disminución del consumo de alcohol, carnes rojas, procesadas y tabaco ^1^.

Cabe destacar que la prevalencia de sobrepeso u obesidad en el departamento de Santander es del 50.1%, superior en los hombres, aunque la obesidad abdominal es más alta en mujeres (46.6% versus 40.11%), el consumo de frutas y verduras es muy bajo (94.9% no alcanza el consumo diario recomendado) ^29^, y se ha documentado desigualdades de género y socioeconómicas en el práctica de actividad física, siendo las mujeres menos activas, especialmente aquellas dedicadas al trabajo no remunerado ^30^. Las desigualdades en los estilos de vida demandan abordajes comprensivos en el que se profundice sobre el papel de las estructuras sociales en la configuración de las decisiones y prácticas en salud, en tanto estas últimas no dependen solamente de la decisión personal ^31^^,^^32^.

Mejorar y propender por la equidad en todos los niveles preventivos en relación con el cáncer de estómago también supone una prioridad para el AMB, máxime cuando se han documentado marcadas desigualdades socioeconómicas en la supervivencia de los pacientes, a pesar de la existencia del sistema “universal” de seguridad social en salud ^33^.

Con relación al cáncer de tiroides, este presenta un aumento en las tasas de incidencia similar a los datos reportados por varias regiones del mundo y del país. En algunos países como Estados Unidos el aumento de las tasas de incidencia se registró hace tres décadas debido al mayor uso de métodos diagnósticos como ecografías, logrando encontrar nódulos muy pequeños que probablemente pasarían desapercibidos por largo tiempo, es decir, el aumento relativo de la incidencia podría estar influenciado por el diagnóstico. 

Finalmente, el comportamiento de las neoplasias hematolinfoides especialmente en las Leucemias con tasas de mortalidad que han duplicado las tasas del quinquenio anterior en nuestra región genera inquietudes sobre cuáles son los factores que han impactado en este aumento marcado en sus tasas de mortalidad. Es importante explorar factores modificables especialmente aquellos que se han asociado con las barreras en la atención de salud de pacientes con cáncer como son factores administrativos, económicos y culturales con demoras en la oportunidad de diagnóstico y tratamiento ^34^.

## Conclusiones

En este estudio se presenta la magnitud del cáncer en el Área Metropolitana de Bucaramanga durante el quinquenio 2008-2012 comparativamente con el quinquenio 2003-2007. Se revelan comportamientos positivos en varios cánceres, que muestran una disminución en la incidencia de un quinquenio a otro o magnitudes inferiores a las del país; sin embargo, resalta la magnitud y la tendencia al aumento del cáncer colorrectal en ambos sexos, así como la alta frecuencia del cáncer de estómago y el aumento de la incidencia por cáncer de tiroides en las mujeres. Asimismo, la mortalidad por cáncer de colon y recto en los hombres y por leucemias en ambos sexos aumenta, lo cual requiere indagaciones posteriores y el fortalecimiento de las medidas preventivas.
